# Peptide-Based [^68^Ga]Ga Labeled PET Tracer for Tumor Imaging by Targeting Tumor-Associated Macrophages

**DOI:** 10.3390/pharmaceutics14112511

**Published:** 2022-11-18

**Authors:** Mingxing Huang, Rang Wang, Mufeng Li, Huawei Cai, Rong Tian

**Affiliations:** Department of Nuclear Medicine, West China Hospital, Sichuan University, 37 Guoxue Alley, Chengdu 610041, China

**Keywords:** gallium-68, radionuclide, peptide, tumor-associated macrophages, probe

## Abstract

Tumor-associated macrophages (TAMs) are known to promote cancer development and metastasis. In this study, a TAMs-targeting peptide named M2pep was selected to investigate the feasibility of [^68^Ga]Ga-labeled M2pep as a noninvasive probe in targeted TAMs imaging. The peptide M2pep was conjugated with 1,4,7,10-tetraazacyclododecane-1,4,7,10-tetraacetic acid (DOTA) and radiolabeled with ^68^Ga. The cellular uptake and binding assay were assessed in M2 macrophages and in the B16F10 cell line. Micro-PET imaging and a biodistribution study were performed on B16F10 tumor-bearing mice. High radiochemical purity [^68^Ga]Ga-DOTA-M2pep (>95%) was prepared and was stabilized in saline and bovine serum at 37 °C for 2 h. In vitro studies demonstrated high uptake of [^68^Ga]Ga-DOTA-M2pep in M2 macrophages, which was effectively blocked by the “cold” M2pep (free peptide). The micro-PET imaging and biodistribution study revealed that [^68^Ga]Ga-DOTA-M2pep reached the tumor site rapidly and showed high accumulation in the tumor at 1 h post-injection. In addition, the probe was rapidly cleared from the blood and mainly excreted via the kidneys, resulting in a high tumor/background ratio. Preclinical studies have shown that [^68^Ga]Ga-DOTA-M2pep specifically targets TAMs and might be a promising molecular probe for the noninvasive visualization of TAMs expression.

## 1. Introduction

The tumor microenvironment (TME) refers to the internal environment, which plays an essential role in tumor genesis and development, promoting tumor growth, metastasis, and drug resistance [1]. Tumor-associated macrophages (TAMs) are derived from peripheral blood monocytes and are the most abundant immune cells within the TME. Monocytes are recruited to the microenvironment and polarized into subtypes of macrophages in response to microenvironmental factors [2,3]. TAMs are classified as M1-like macrophages with antitumor properties and M2-like macrophages with tumor-promoting properties [4,5]. Nevertheless, TAMs usually display the M2 phenotype and function by activating tumor cell proliferation and promoting angiogenesis and metastasis. Several studies have reported the correlation between M2 TAMs infiltration and cancer growth, metastasis, and poor prognosis [6,7]. Monitoring the infiltration of M2 TAMs in the TME enables the prediction of patient prognosis and can be used to guide clinical treatment. Tumor biopsy is the gold standard to assess the expression or density of M2 TAMs. However, the biopsy is invasive and is limited by sampling errors and interobserver variability. In addition, some tumor sites are not accessible for biopsy, and the tumor specimen may not illustrate the actual tumor burden due to heterogeneity. Therefore, noninvasive imaging modalities can be effectively used to provide valuable information.

Traditionally, the use of noninvasive imaging such as CT, US, and MRI as diagnostic tools was limited to detecting structural abnormalities and evaluating tumor volume and morphology. Noninvasive functional imaging techniques, including PET and SPECT, provide valuable biologic information with high specificity and sensitivity. These techniques can be combined with CT or MRI as PET/CT or PET/MRI, allowing for noninvasive visualization of the disease process and evaluation of the therapeutic response [8,9,10]. Hence, TAMs-specific targeted radionuclide imaging can improve disease diagnosis, therapy selection, and treatment monitoring [11,12].

Several studies have developed macrophage-specific radiotracers to assist in tumor diagnosis and therapy. The radiotracers bind to the most distinctive surface markers of M2 macrophages, including the macrophage mannose receptor (MMR) CD206, the macrophage scavenger receptor CD163, and the folate receptor beta (FR-β) [13,14,15]. However, these membrane receptors are also highly expressed on normal macrophages in the mononuclear phagocyte system organs. M2 macrophage-binding peptide (M2pep) was reported to have high selectivity and efficient targeting ability to M2 macrophages [16,17]. Several studies have demonstrated that M2pep-modified M2 TAMs-targeting nanoparticles were successfully used in tumor therapy by delivering drugs or inhibitors to TME. TMEs are polarized from the M2 phenotype to the M1 phenotype, thus stimulating the inflammatory response and suppressing tumor growth [18,19,20]. ^68^Ga has a short half-life and is compatible with the pharmacokinetics of the radiolabeled peptide. In addition, it can be routinely obtained from a ^68^Ge/^68^Ga generator. The value of ^68^Ga as a PET imaging agent in clinical research has gradually been acknowledged.

In this study, M2pep was functionalized with the chelator DOTA to obtain DOTA-M2pep and then radiolabeled with ^68^Ga. This study aimed to evaluate the in vitro and in vivo characteristics of [^68^Ga]Ga-DOTA-M2pep and investigate the attributes of [^68^Ga]Ga-DOTA-M2pep as a noninvasive PET/CT probe for tumor imaging by targeting M2 TAMs.

## 2. Materials and Methods

### 2.1. Synthesis of DOTA-M2pep and Radiolabeling

In our study, M2pep (YEQDPWGVKWWY) and DOTA-M2pep (1,4,7,10-tetraazacyclododecane-1,4,7,10-tetraacetic acid-YEQDPWGVKWWY) were synthesized using solid-phase chemistry by Jie Tai Biotechnology (Nanjing, China). High-performance liquid chromatography (HPLC) and electrospray ionization mass spectrometry (ESI-MS) were used to detect the purity and mass of the radiolabeled precursor. Furthermore, nuclear magnetic resonance spectroscopy (NMR spectroscopy) was used to determine the structure of DOTA-M2pep. [^68^Ga]Ga was obtained from a ^68^Ge/^68^Ga-generator (ITM, Munich, Germany) by eluting with 0.1 M HCl solution. The rest of the chemicals were purchased from commercial suppliers.

DOTA-M2pep (80 μg) was dissolved in 200 μL of purified water in a small vial. About 200 μL of [^68^Ga]GaCl_3_ (44.4–59.2 MBq) was added, and 1 M sodium acetate buffer was used to adjust the final pH to 4.0–4.5. After incubation in a water bath at 55 °C for 30 min, the radiolabeling reaction was completed, and the mixture was collected. Radio-HPLC was performed to confirm the radiochemical purity of [^68^Ga]Ga-DOTA-M2pep. The mobile phase was composed of solvent A, acetonitrile (ACN) solution, and solvent B, 0.1% trifluoroacetic acid (TFA) in water. The gradient solvent system was set up as follows: 0–8 min, 70% A and 30% B; 8–15 min, 100% A and 0% B; 15–17 min, 5% A and 95% B; 17–20 min, 95% A and 5% B at a flow rate of 1.0 mL/min. To investigate the in vitro stability, 3.7 MBq [^68^Ga]Ga-DOTA-M2pep (100 μL) was incubated in the same volume of phosphate-buffered saline (PBS, pH = 7.4) or bovine serum at 37 °C, respectively. The radiochemical purity was assessed at 15, 30, 60, and 120 min. All experiments were performed in triplicate.

### 2.2. Cell Culture and Animal Model

Bone-marrow-derived macrophages (BMDMs) were cultured from bone-marrow cells according to an established protocol [18]. The bone marrow (BM) cells were harvested from the femurs and tibias of C57BL/6 mice (male, 6–8 weeks old). After centrifugation, the bone marrow cells were cultured in RPMI medium 1640 supplemented with macrophage colony-stimulating factor (M-CSF, Peprotech, Waltham, MA, USA, 40 ng/mL) and seeded in a cell culture dish. On day 3, the medium was half-replaced with fresh medium. On day 6, immature macrophages were harvested and cultured further for 48 h in a new medium with IL-4 (Peprotech, 20 ng/mL) to induce macrophage polarization toward M2-like macrophages.

B10-F10 (mouse melanoma) cells were obtained from the Cell Bank of the Chinese Academy of Sciences, and cultured in Dulbecco modified Eagle medium (DMEM, Hyclone, Waltham, MA, USA) supplemented with 10% fetal bovine serum (FBS, Gibco, Waltham, MA, USA), 100 U/mL penicillin and 100 μg/mL streptomycin. All cells were cultured at 37 °C, 5% CO_2_, and 95% humidity.

The male C57B/L6 mice (6–8 weeks old, 20–25 g) were purchased from HFK bioscience (Beijing, China) and divided randomly into the experimental and blocking groups. All the studies were approved by the experimental animal ethics committee of Sichuan University and were conducted in compliance with the institutional guidelines for the care and use of laboratory animals. The C57B/L6 mice were injected subcutaneously with 5 × 10^6^ B16-F10 cells in 100 µL of PBS into the forelimb of the animals under anesthesia with isoflurane. After 7–10 days, the B16F10 tumor-bearing mice were established and prepared for imaging and biodistribution when xenografts grew to 500–1000 mm^3^.

### 2.3. In Vitro Uptake and Binding Assay

M2 macrophages and B16-F10 cells were pre-seeded in 6-well microplates with a density of 5 × 10^5^ cells/well to estimate the in vitro binding affinity of [^68^Ga]Ga-DOTA-M2pep. After overnight incubation, the medium was removed from the wells and the cells were washed twice with PBS. Then, 74 KBq [^68^Ga]Ga-DOTA-M2pep was added to each well, followed by incubation for 15 min, 30 min, 60 min, and 120 min. Subsequently, the cells were washed three times with PBS, the supernatant of each well was collected, and cells were harvested with 1 M sodium hydroxide solution (0.5 mL). Both the supernatant and the cell suspension were collected, and the radioactivity was evaluated using an automatic γ-counter (PerkinElmer Wizard, Shelton, CT, USA).

A competitive binding assay was also performed on the M2 macrophages and B16-F10 cells. 74 KBq [^68^Ga]Ga-DOTA-M2pep and [^68^Ga]GaCl_3_ were added to the experimental and the blank wells respectively. For the blocking experiment, the cells were pretreated with an excess of free M2pep (10 mM) for 60 min, followed by radiotracer incubation. After 60 min of incubation at 37 °C, the cells were collected and measured using the above-mentioned uptake assay. All the experiments were performed in triplicate.

### 2.4. Micro-PET Imaging

PET scans were performed using a micro-PET/CT scanner (Inviscan, IRIS, Paris, France). [^68^Ga]Ga-DOTA-M2pep (3.7 MBq) was administered to the B16F10 tumor-bearing mice (*n* = 4) via the tail vein. At 30 min, 1 h, and 2 h post-injection, the tumor-bearing mice were imaged with the micro-PET/CT scan under anesthesia with 2% isoflurane. Each scan was completed within 15 min. For the blocking scans, B16F10 tumor-bearing mice (*n* = 3) were pretreated with free M2pep (10 mM) in 100 μL saline by intravenous injection 60 min before administration of [^68^Ga]Ga-DOTA-M2pep (3.7 MBq). Micro-PET/CT scans were acquired at 60 min post-injection as described above. The obtained images were reconstructed using three-dimensional ordered-subset expectation maximization (3D OSEM) and then processed using Osirix MD. Regions of interest (ROIs) were manually drawn over the tumor regions and organs, and quantitative analyses were calculated. Immunofluorescence staining was performed 24 h after micro-PET scan to determine the [^68^Ga]Ga-DOTA-M2pep targeting efficiency. The harvested tumors were fixed with paraformaldehyde for 24 h, then were frozen and sectioned at 10 μm, followed by treatment with Fitc-M2pep, and staining with M2 TAMs marker PE-CD206 antibody and DAPI. The localization of Fitc-M2pep and PE-CD206 signals was observed with a confocal laser scan microscope (Zeiss, Jena, Germany).

### 2.5. Biodistribution Studies

The B16F10 tumor-bearing mice (*n* = 3) were intravenously administered [^68^Ga]Ga-DOTA-M2pep (1.11 MBq) for biodistribution studies. At 5, 15, 30, 60 min, and 2 h post-injection, mice were euthanized, and the organs of interest, including the blood, heart, lungs, liver, kidneys, spleen, stomach, intestine, muscle, bone, brain, and tumor, were harvested. The samples were then weighed and counted by an automatic γ-counter (PerkinElmer Wizard, Shelton, CT, USA). In the blocking group, the B16F10 tumor-bearing mice (*n* = 3) were pretreated with free M2pep (10 mM) in 100 μL saline by intravenous injection 60 min before administration of [^68^Ga]Ga-DOTA-M2pep (1.11 MBq). The organs of interest were harvested, weighed, and counted as described above at 60 min post-injection. The percentage of tracer uptake was calculated as the percentage of injected dose per gram of tissue (%ID/g), which was decay-corrected to the start time of counting. The percentage reflected the activity associated with tissues per organ weight per actual injected dose.

### 2.6. Statistical Analysis

Statistical analysis was performed by OriginPro software. The reported values were presented as mean ± standard deviation. In the unpaired t-test, *p*-values < 0.05 were considered statistically significant.

## 3. Results

### 3.1. Synthesis, Radiolabeling and Stability

**Figure 1:**
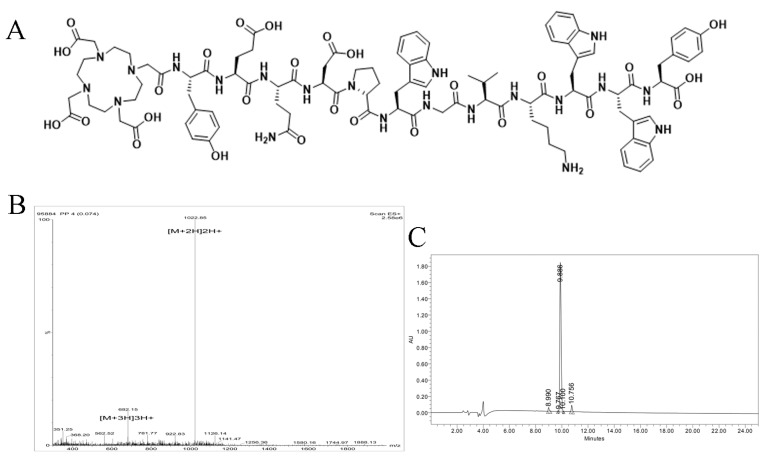
(**A**) The structural formula of DOTA-M2pep. Mass spectrometry (**B**) and HPLC (**C**) results for DOTA-M2pep.

**Figure A1:**
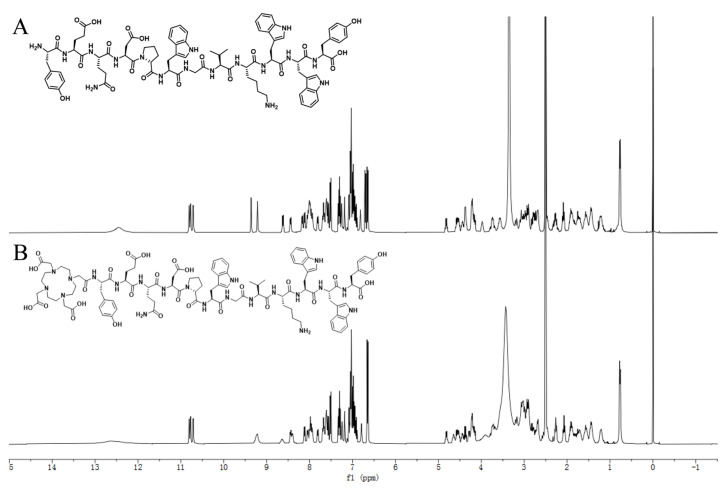
^1^H NMR spectrum of M2pep (**A**) and DOTA-M2pep (**B**) in DMSO. Δ = 6.5~8.5 ppm (M2pep).

The DOTA-M2pep was successfully synthesized and its structure is shown in Figure 1A. The molecular weight (*m*/*z* C_99_H_127_N_21_O_27_ [M]^+^ = 2043.68) was determined by ESI-MS (Figure 1B), and HPLC (Figure 1C) showed that the chemical purity of DOTA-M2pep was greater than 95%. The ^1^H NMR spectrum of M2pep and DOTA-M2pep are demonstrated in Figure A1, which identified the same characteristic peak.

**Figure 2:**
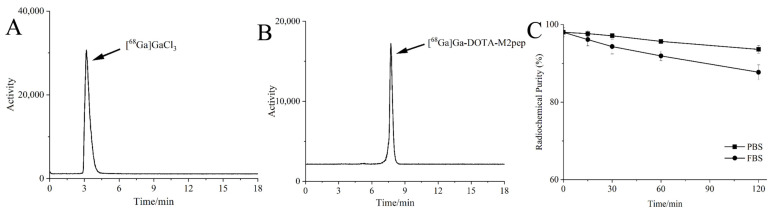
(**A**) Radio-HPLC of [^68^Ga]GaCl_3_. (**B**) Radio-HPLC of [^68^Ga]Ga-DOTA-M2pep, showing a radiochemical purity of over 95%. (**C**) Stability of [^68^Ga]Ga-DOTA-M2pep in phosphate-buffered saline (PBS) and fetal bovine serum (FBS) over 2 h.

[^68^Ga]Ga-DOTA-M2pep was generated with a radiochemical purity of 98 ± 1.15% under heating at 55 °C for 30 min and used without further purification. The specific activity and the molar activity of the radiolabeled peptide were 0.24–0.33 MBq/μg and 0.20–0.27 MBq/μmol, respectively. As displayed in Figure 2, the retention time of [^68^Ga]Ga-DOTA-M2pep on radio-HPLC was 8.3 min. The [^68^Ga]Ga-DOTA-M2pep was stable, and the radiochemical purity was still over 90% and 85% in PBS and FBS after 2 h of incubation, respectively.

### 3.2. In Vitro Uptake and Binding Assay

**Figure 3:**
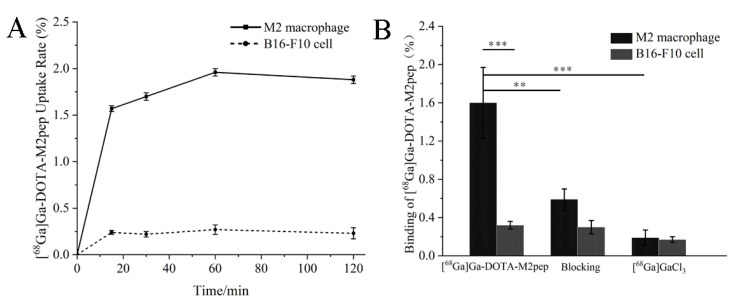
(**A**) Uptake of [^68^Ga]Ga-DOTA-M2pep in macrophages and B16F10 cells. (**B**) Competitive binding assay of [^68^Ga]Ga-DOTA-M2pep in macrophages and B16F10 cells (** *p* < 0.01, *** *p* < 0.001).

**Figure A2:**
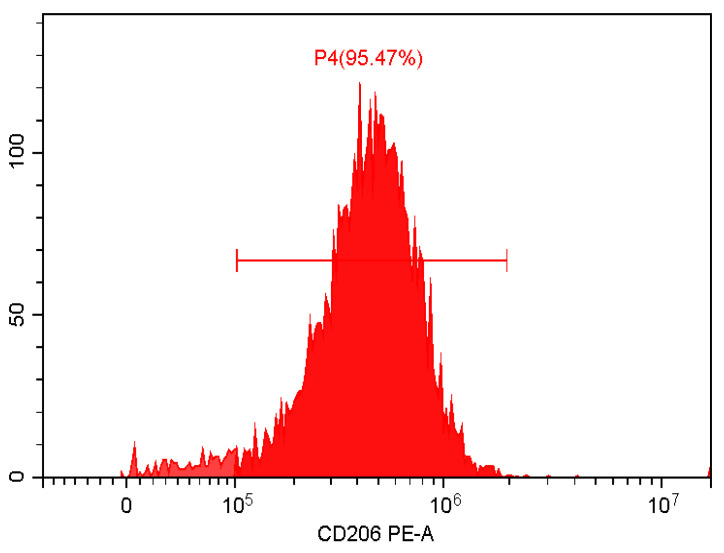
Identification of in vitro polarization of M2 macrophages. Bone-marrow-derived macro phages (BMDMs) were incubated with IL-4 (20 ng/mL) on day 6 for 48 h. Cells were collected and stained with PE-CD206. The positive percentage of PE-CD206 were measured by flow cytometry.

Bone-marrow-derived macrophages were obtained and successfully polarized to the M2 phenotype (Figure A2). Significant differences in cell uptake kinetics of [^68^Ga]Ga-DOTA-M2pep were demonstrated between M2 macrophages and B16F10 cells (Figure 3A). Furthermore, [^68^Ga]Ga-DOTA-M2pep showed a time-dependent accumulation in M2 macrophage. The uptake of [^68^Ga]Ga-DOTA-M2pep was rapid and almost saturated within 30 min, and the highest uptake of 1.96 ± 0.04% was achieved at 1 h. In contrast, B16F10 cells showed a low cell uptake during incubation (1.96 ± 0.04% vs. 0.27 ± 0.05%, *p* < 0.001). The M2 macrophages were subjected to a competitive binding assay with non-radiolabeled M2pep revealing a significant decrease in cell uptake in blocking studies. The findings indicated that the binding of [^68^Ga]Ga-DOTA-M2pep to M2 macrophage was blocked by M2pep (1.6 ± 0.37% vs. 0.59 ± 0.11%, *p* = 0.0029) (Figure 3B).

### 3.3. Micro-PET Imaging

**Figure 4:**
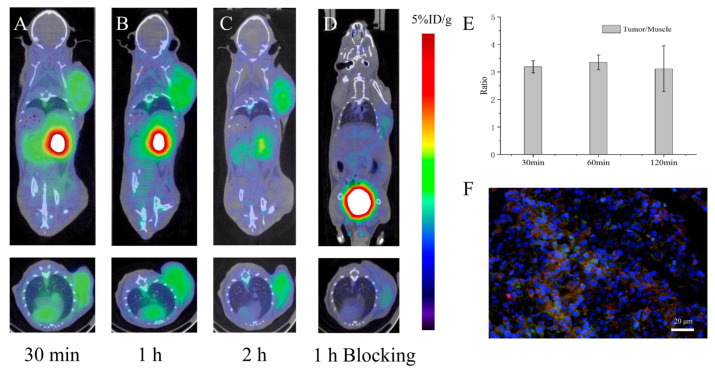
(**A**–**C**) PET images of the B16F10 tumor-bearing mice 30 min, 1 h, and 2 h after injection of [^68^Ga]Ga-DOTA-M2pep, respectively (*n* = 4). (**D**) PET images of the blocking groups 1 h after injection of [^68^Ga]Ga-DOTA-M2pep. The arrows indicate the tumor regions (*n* = 3). (**E**) Tumor-to-muscle (T/M) ratios in B16F10 tumor-bearing mice at different time points. (**F**) Immunofluorescence of tumor sections. Tumor sections were stained with FITC-M2pep (green), CD206 (red), and DAPI (blue) after micro-PET imaging.

Micro-PET scans were performed at 30 min, 1 and 2 h following injection of [^68^Ga]Ga-DOTA-M2pep. The coronal and transverse PET/CT images showed an obvious uptake of the radiolabeled tracer in tumor tissues (Figure 4A–C). The blocking scan demonstrated mild radiotracer uptake at 1 h post-injection of [^68^Ga]Ga-DOTA-M2pep (Figure 4D). Significant accumulation of [^68^Ga]Ga-DOTA-M2pep was also visualized in the kidneys and bladder over time, while low accumulation was detected in the liver, which indicated that [^68^Ga]Ga-DOTA-M2pep was mainly excreted via the urinary system. Quantitative analysis revealed that the uptake values of [^68^Ga]Ga-DOTA-M2pep in B16F10 tumor at 30 min, 1, and 2 h were 2.72 ± 0.07, 2.93 ± 0.20, and 2.47 ± 0.13 %ID/g, respectively. The tumor/muscle ratios at 30 min, 1 h, and 2 h were 3.19 ± 0.22, 3.35 ± 0.27, and 3.12 ± 0.83, respectively (Figure 4E). Injection of a large excess of free M2pep resulted in a significant reduction to 1.23 ± 0.13 %ID/g in tumors (*p* < 0.001), suggesting that M2pep blocked the specific targeting of [^68^Ga]Ga-DOTA-M2pep to TAMs. In vitro immunofluorescence staining confirmed the expression of M2 TAMs in B16F10 tumors and the specificity of [^68^Ga]Ga-DOTA-M2pep targeting M2 TAMs (Figure 4F).

### 3.4. Biodistribution Studies

**Figure 5:**
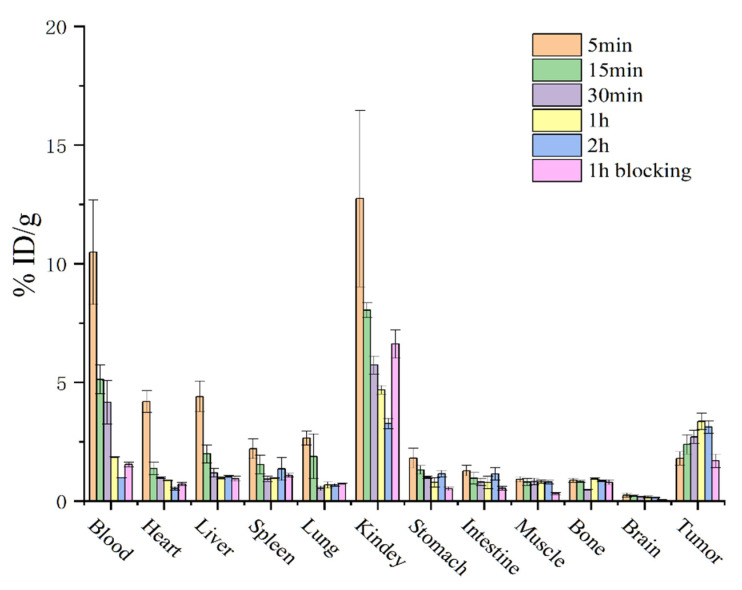
Biodistribution of [^68^Ga]Ga-DOTA-M2pep in B16F10 tumor-bearing mice (*n* = 3) at different time points. In the blocking group, the biodistribution was examined in B16F10 tumor-bearing mice (*n* = 3) at 60 min post-injection of [^68^Ga]Ga-DOTA-M2pep. Data are presented as %ID/g and are expressed as mean ± SD.

The biodistribution of [^68^Ga]Ga-DOTA-M2pep at different time points in B16F10 tumor-bearing mice is shown in Figure 5, which was consistent with the micro-PET imaging results. Fast clearance of [^68^Ga]Ga-DOTA-M2pep from blood was observed, with a blood uptake of 10.48 ± 2.20 %ID/g at 5 min and 1.84 ± 0.02 %ID/g at 60 min after injection. The highest uptake of tracer was found in the kidney, which peaked at 5 min (12.74 ± 3.71 %ID/g) and then decreased by more than 50% at 1 h. A relatively low accumulation of tracer was found in the liver, with an uptake value of 0.96 ± 0.02 %ID/g at 1 h post-injection. In addition, relatively lower levels of activities were detected in the brain, stomach, intestines, spleen, and bone. Moreover, the uptake of [^68^Ga]Ga-DOTA-M2pep in tumors was highest at 1 h, with an uptake value of 3.35 ± 0.34 %ID/g, which then decreased to 3.12 ± 0.27 %ID/g at 2 h. In the blocking group, the uptake of [^68^Ga]Ga-DOTA-M2pep decreased in B16F10 tumors from 3.35 ± 0.34 %ID/g to 1.69 ± 0.28 %ID/g (*p* < 0.01). In addition, the activity in the blood and tissues were found to be similar in normal and blocking groups at 1 h post-injection of [^68^Ga]Ga-DOTA-M2pep, which demonstrated the similar metabolic features of the tracer in vivo (Figure 5).

## 4. Discussion

In the present study, the radionuclide labeling M2pep was developed, and the molecular probe [^68^Ga]Ga-DOTA-M2pep specifically targeting M2 TAMs was obtained. The feasibility of [^68^Ga]Ga-DOTA-M2pep for PET imaging of TAMs was investigated by in vivo and in vitro experiments.

TAMs play an essential role in tumor progression by stimulating tumor angiogenesis, invasion, and metastasis [21]. Therefore, TAMs are considered a new potential target in tumor imaging and treatment. PET imaging by targeting TAMs not only allows noninvasive visualization of tumor lesions but also confirms the expression of TAMs in tumors quantitatively, thereby guiding individualized therapy. The radionuclide ^68^Ga was selected to label M2 TAMs-specific targeted peptide M2pep. High radiochemical purity [^68^Ga]Ga-DOTA-M2pep was produced, which did not require further purification and remained stable in PBS and FBS for 2 h. The labeling process of [^68^Ga]Ga-DOTA-M2pep is simple and fast compared to the ^11^C or ^18^F labeling of antibody fragments. ^11^C and ^18^F labeling ligands involve complicated radiochemical synthesis and lower radiochemical yield, limiting their use as radiotracers in PET imaging targeting TAMs [22,23]. In addition, the radionuclides ^11^C and ^18^F require an onsite cyclotron for production, resulting in higher operating costs.

Cell uptake and block experiments were performed with M2 macrophages and B16F10 cells to verify the specificity and cellular binding kinetics of the probe. [^68^Ga]Ga-DOTA-M2pep binds preferentially to M2 macrophages over B16F10 cells. The former was significantly decreased by adding excess non-radiolabeled M2pep, indicating that [^68^Ga]Ga-DOTA-M2pep specifically targets M2 macrophages, which is in accordance with the literature on M2pep and M2 macrophages [18,24].

Subsequently, the feasibility of [^68^Ga]Ga-DOTA-M2pep to evaluate M2 TAMs-specific targeting was investigated in B16F10 tumor-bearing mice. The in vivo micro-PET imaging and biodistribution study indicated that [^68^Ga]Ga-DOTA-M2pep had high specificity and rapid excretion. [^68^Ga]Ga-DOTA-M2pep easily reached the tumor sites and was rapidly cleared from the normal tissues, resulting in superior tumor-to-normal tissue contrast. The tumors could be visualized at 1 h after injection of [^68^Ga]Ga-DOTA-M2pep, which is shorter than the current TAMs targeting antibodies or macromolecular polymer probes ^18^F-fluorobenzoate (FB)-anti-MMR sdAb and [^89^Zr]Zr-AI-HDL [25,26]. Due to the slow metabolism of antibodies and macromolecular polymers in vivo, the optimal imaging times of ^18^F-fluorobenzoate (FB)-anti-MMR sdAb and [^89^Zr]Zr-AI-HDL were 3 h and 24 h, respectively. In the past, Xavier et al. utilized ^68^Ga labeling single-domain antibody fragments to target CD206 with PET imaging [27]. However, [^68^Ga]Ga-NOTA-anti-MMR-sdAb showed higher uptake in tumors, liver, and spleen of wild-type tumor-bearing mice compared to CD206 knock-out mice, which could limit the application of that molecular probe.

[^68^Ga]Ga-DOTA-M2pep has a high specificity for TAMs, but the retention was relatively low.

## 5. Conclusions

The novel ^68^Ga-labeled peptide ([^68^Ga]Ga-DOTA-M2pep) was successfully prepared with high radiolabeling purity. Preclinical studies indicate that [^68^Ga]Ga-DOTA-M2pep can specifically target M2 macrophages and is a promising tracer for the noninvasive visualization of TAMs expression.

## Data Availability

The data are available on reasonable request from the corresponding authors.
